# The predictive value of the change of the number of pixels under different CT value intervals in the CT-occult central lung squamous cell carcinoma and squamous epithelial precancerous lesions

**DOI:** 10.1186/s12890-023-02732-w

**Published:** 2023-11-03

**Authors:** Jiaming Zhou, Bijun Yu, Peng Guo, Shi Wang

**Affiliations:** Department of Endoscopy, Zhejiang Cancer Hospital, Hangzhou, China

**Keywords:** Lung cancer, Lung computed tomography, CT value, Number of pixels, CT-occult, Central, Lung squamous cell carcinoma, Squamous epithelial precancerous lesions

## Abstract

**Background:**

Due to the fact that the CT-occult central lung squamous cell carcinoma and squamous epithelial precancerous lesions.

(CT-occult CLSCC and SEPL) cannot be detected by lung CT screening, early and timely diagnosis of central lung cancer becomes very difficult, which directly affects the prognosis of patients.

**Methods:**

We retrospectively review medical records of patients at the Zhejiang Cancer Hospital and enrolled 41 patients with the CT-occult CLSCC and SEPL and 48 patients without the CT-occult CLSCC and SEPL. We compare the clinical characteristics, imaging features and Changes in the number of pixels under different CT value intervals of patients with and without the CT-occult CLSCC and SEPL and we perform univariate and multivariate logistic regression analysis to explore independent factors for the CT-occult CLSCC and SEPL in the patients.

**Results:**

We demonstrate that pack-years ≥ 20 (OR: 3.848, 95% CI: 1.086 ~ 13.633), the number of pixels change of CT value in interval [-850 ~ -750HU] (OR: 5.302, 95% CI: 1.122 ~ 25.057) and in interval [-900 ~ -850HU] (OR: 3.478, 95% CI: 1.167 ~ 10.365) are independently associated with the CT-occult CLSCC and SEPL in the patients. Ultimately, the logistic model obtained is statistically significant (*p* < 0.05) and an area under the ROC curve is 0.776 (95% CI: 0.682–0.870). The sensitivity of this model is 90.2% and the specificity is 52.1%.

**Conclusion:**

The results of this study indicate that in the CT value range [-950 ~ -750HU], when the total number of lung pixels tend to increase towards the region with high CT value, the probability of the occurrence of CT-occult CLSCC and SEPL lesions also increases. Meanwhile, these results have guiding significance for the further study of radiomic.

## Introduction

Primary bronchial lung cancer, referred to as lung cancer (LC) has the highest incidence and mortality of malignant tumors worldwide [1, 2]. Low-dose computed tomography (LDCT) is the best screening method for lung cancer at present.According to the results of the National Lung Cancer Screening Trial in the United States, LDCT screening could reduce the lung cancer death rate of the high-risk population by 20%, all-cause mortality was 6.7% lower than in the control group [3]. Since 2005, our country has carried out a number of national major public health service projects including lung cancer screening in succession, and great progress has been made in screening, diagnosis and treatment of lung cancer [4, 5]. However, the early diagnosis of LC is still insufficient [6, 7]. Meanwhile, as for the bronchial mucosal epithelial lesions represent by CT-occult lung cancer, although it is reported in the literature as early as 1998, as far as we know, its early diagnosis is still troubling clinicians up to now [8].

The concept of radiomics is proposed by Gillies et al. in 2010, it could use computer technology to deeply mine massive medical image data from the perspective of images, extract various quantitative features efficiently and objectively improve the accuracy of diagnosis and make predictions at the same time through detailed quantitative analysis now [9]. Meanwhile, due to the assisting diagnosis of artificial intelligence, doctors can detect more pulmonary nodules with a diameter of less than 5 mm [10]. However the study of lung tumors by imaging is still limited to the area of tumors and suspected tumors [11, 12]. At present, the study of whole lung CT radiomic mainly focuses on the prediction and diagnosis of radiation pneumonia [13, 14]. Therefore, this study is aimed to explore the value of the CT radiomic in predicting the CT-occult central lung squamous cell carcinoma and squamous epithelial precancerous lesions (CT-occult CLSCC and SEPL) by analyzing the whole lung.

## Patients and methods

### Patients selection and data collection

This was a retrospective and observational study, approved by the Biomedical Ethics Review Committee of the Zhejiang Cancer Hospital, Hangzhou, China. A total of 89 patients with peripheral lung adenocarcinoma less than 3 cm in diameter were selected as the study objects admitted to the Department of Endoscopy from January 2021 to December 2021, including 41 patients with the CT-occult CLSCC and SEPL and 48 patients without the CT-occult CLSCC and SEPL. Data on each patient was retrospectively extracted from the electronic medical records, including baseline clinical characteristics, the initial blood tumor markers laboratory examinations and CT imaging when diagnosed newly.

All the 89 patients were male and the age of patients in both groups ranged from 49 to 70. All the patients were workers. None of the 89 patients were complicated with chronic obstructive pulmonary disease (COPD) and no signs of acute inflammation were found on lung CT and bronchoscopy. Smoking levels were expressed in pack-years calculated by multiplying the number of packs of cigarettes consumed per day by the number of years smoked and a history of heavy smoking was defined as equal or greater than 20 pack-years.

In this study, a total of 9 (smoking pack-years ≥ 20, CEA, CA125, CA199, other nodules except the first nodules, focal fibrosis, bulla, CT value interval distribution 1 and 2) independent variables were involved in regression analysis to determine the risk factors for the occurrence of the CT-occult CLSCC and SEPL, and the sample size was 5–10 times the number of independent variables. The CT-occult CLSCC and SEPL were discovered by bronchoscopy. The CT-occult CLSCC and SEPL as the dependent variable included squamous cell carcinoma, moderate to severe squamous epithelial dysplasia, and mild to moderate squamous epithelial dysplasia. Diagnosis of LC and the CT-occult CLSCC and SEPL was performed in accordance with the WHO classification of tumours of the lung, pleura, thymus and heart 4th 2015 [15].

### Artificial intelligence processing of CT

The images of lung CT were collected using Siemens Somatom Definition Flash. The patient was in a conventional supine position with arms raised above his head and breath held once after deep inspiration. The scanning range was from the thoracic entrance to the lower level of the costal diaphragm Angle, including the whole lung. The scanning parameter tube voltage was 120 kV, the tube current was modulated automatically (150-200mAs), and the layer thickness was 5 mm. Lung window (window width 1600HU, window level -450HU), and 1 mm thin layer reconstruction, Mediastinal window (window width 350HU, window position 20HU).

**Fig. 1:**
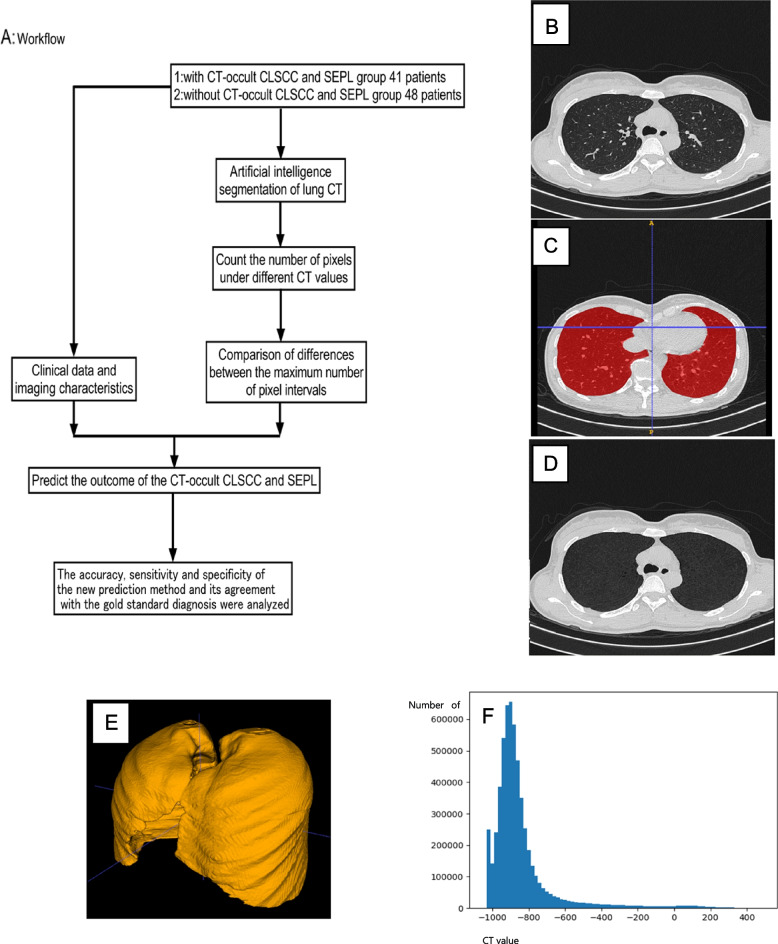
Artificial intelligence processing of CT. **A **Research workflow. **B**-**D **The original CT scan of the lung, Deep learning convolutional neural network was used to remove pulmonary vessels and mask images after segmentation. **E **The two dimensional mask sequence is combined into a three dimensional mask sequence lung image. **F **Pixel distribution of different CT values after 3D combination of mask images after lung segmentation, CT value interval is [-1000,0HU]

The region of interest (ROI) of lung CT set as bilateral normal lung tissue, python was used as the programming language. Simple ITK library was used to read and write Dicom files, and opencv library and keras library were used for image algorithm processing and model training. Radiographic annotation used the open source ITK-SNAP and simultaneously used ITK-SNAP to map the three-dimensional lung. Then, convolution neural network deep learning model obtained by training unet with open source data set was used to segment lung data, and the target nodules, pulmonary hilum, clear pulmonary vessels, bronchus, other clear pulmonary nodules and bullosa were separated. At the same time, the pixel distribution of lung tissue with CT interval of [-1000,0HU] was described (Fig. 1).

Each pixel in the CT image would correspond to a different CT value. When the property of lung tissue changed, the CT value of this part of lung tissue would also change correspondingly, so the number of pixels under different CT values would also change correspondingly. Meanwhile, the total number of pixel also changed in the corresponding CT value interval. The total number of pixels in a person's lung varied greatly depending on height and weight, and most of the pixels in lung CT were concentrated in the range of [-950 ~ -750HU]. Therefore, this study did not compare the specific number of pixels, but only compared the size of the sum pixels between the two specific adjacent CT value intervals.

In this study, the CT value interval distribution 1: The interval distribution of CT values was established at intervals of 100HU from -950HU to -50HU and the CT value interval distribution 2: The interval distribution of CT values was established at intervals of 50HU from -950HU to 0HU. According to the calculation of the number of CT pixels in 89 patients, in distribution 1, the CT value interval with the largest number of pixels in the study object was [-950 ~ -850HU] or [-850 ~ -750HU] and in distribution 2, the CT value interval with the largest number of pixels in the study object was [-950 ~ -900HU] or [-900 ~ -850HU] respectively.

### Statistical analysis

The analysis was performed with SPSS 24.0 (SPSS Inc. Chicago, IL, USA). Qualitative variables were expressed as frequencies and percentages, and quantitative variables were expressed as the median and interquartile range (IQR). For qualitative variables, we used Fisher’s exact test or X2 test depending on the data. The normality test was performed using Shapiro Wilk. Non-normality quantitative variables were compared by Mann–Whitney U test. Binary logistic regression analysis was performed to identify the association between clinical factors and the CT-occult CLSCC and SEPL and odds ratios (OR) and 95% confidence intervals (95% CIs) were estimated. Firstly, regression analysis was conducted for each independent variable separately, then all independent variables with *P* < 0.05 were included in multiple regression analysis together and the joint predicted value was obtained. Receiver operating characteristic (ROC) curves with an area under the curve (AUC) were constructed to determine the sensitivity and the specificity of model. In this study, *P* < 0.05 was considered as statistically significant.

## Results

### Lesion features in patients with the CT-occult CLSCC and SEPL

**Fig. 2:**
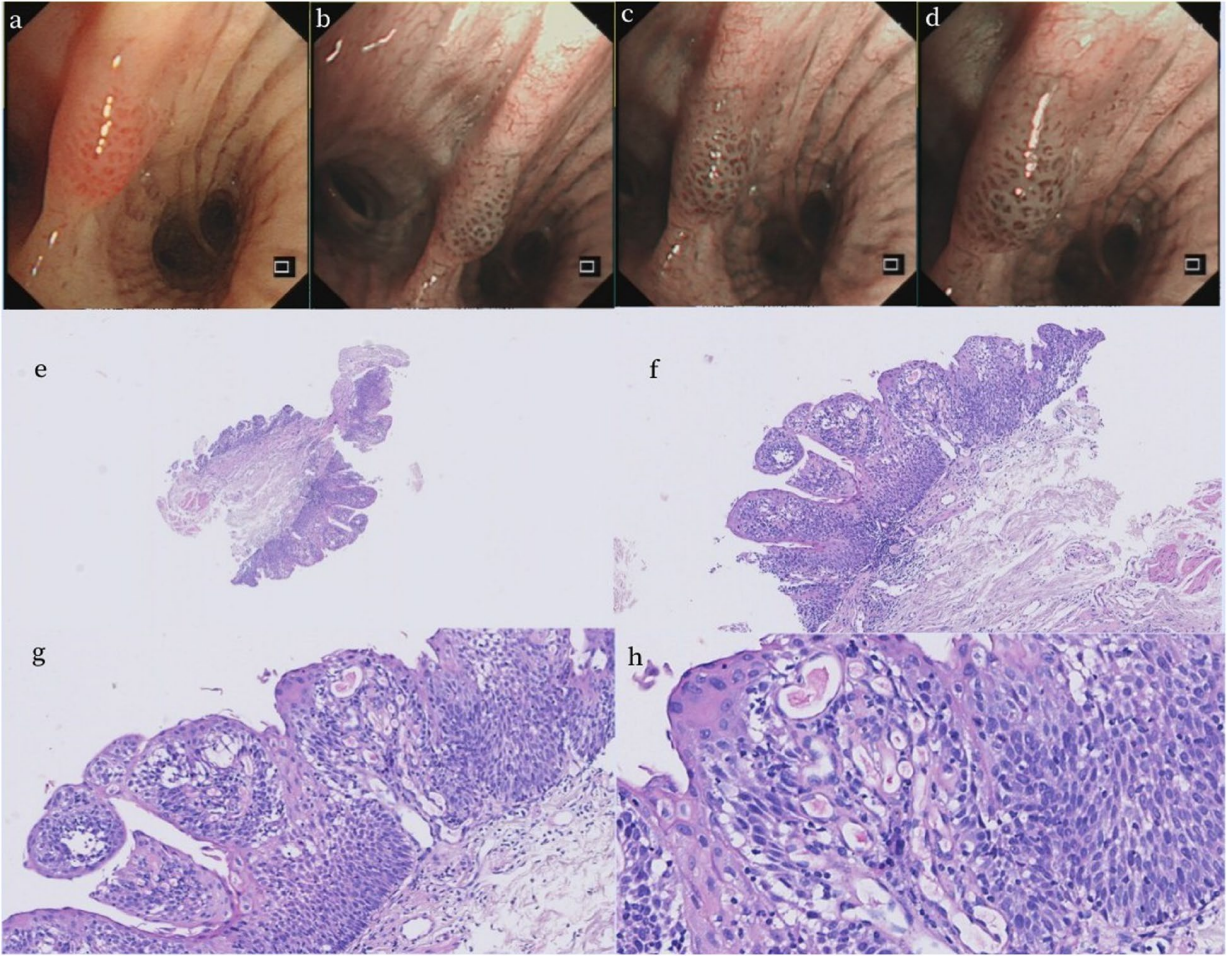
Bronchoscopic and pathological images of a CT-occult lesion. The patient was a 65-year-old male with a history of heavy smoking for more than 40 years. He was admitted to the hospital due to pulmonary nodules found in physical examination, and CT occult bronchial mucosal lesions of the central airway were found during bronchoscopy. **a** Conventional white light mucosal lesion morphology under bronchoscopy. **b**-**d** Mucosal vascular morphology under narrow-band imaging mode under bronchoscopy. **e**–**h** Pathological section images magnified 40 times, 100 times, 200 times and 400 times, respectively, Pathological diagnosis was mucosal squamous epithelial papillary hyperplasia with local mild dysplasia

The common pathological types of the CT-occult CLSCC and SEPL was mild to moderate squamous epithelial dysplasia (24/41, 58.6%) more than moderate to severe squamous epithelial dysplasias(11/41 26.8%) and squamous carcinoma (6/41 14.6%) in this study (Fig. 2).

### Comparison between the patients with and without the CT-occult CLSCC and SEPL

The proportion of ex-or-current smokers, the level of smoking pack-years ≥ 20 and the levels of CEA and CA125 in the CT-occult CLSCC and SEPL group were significantly higher than those in the control group respectively. There were no statistical differences in the levels of CA199 and in the presence of other nodules except the first nodules, focal fibrosis and bulla between the two groups respectively. Among the patients in the interval distribution 1, the proportion of patients in the CT-occult CLSCC and SEPL group in interval [-850 ~ -750HU] (26.8% vs. 6.2%, *p* < 0.05) was higher than that in the control group and in the interval distribution 2, the proportion of patients in the CT-occult CLSCC and SEPL group in interval[-900 ~ -850HU] (82.9% vs. 50%, *p* < 0.05) was higher than that in control group (Table 1).

**Table 1:** Clinical characteristics and imaging features in the patients with and without the CT-occult CLSCC and SEPL

	CT-occult CLSCC and SEPL	Non CT-occult CLSCC and SEPL	*p* Value
	*n* = 41	*n* = 48	
Patient characteristics, number(%) or median(IQR)			
Sex (men)	41/41 (100.0%)	48/48 (100.0%)	
Age, years	62(10)	60.05(10)	0.083
Ex-or-current smokers	35/41(85.4%)	30/48(62.5%)	0.015
Pack-years ≥ 20	35/41(85.4%)	30/48(62.6%)	0.015
Laboratory examinations, median (IQR)			
CEA(ng/ml)	1.97(2.57)	1.285(1.62)	0.046
CA125(U/ml)	10.4(8.85)	7.95(5.5)	0.011
CA199(U/ml)	8.63(10.26)	9.99(7.32)	0.633
Radiological examinations, number (%)			
Other nodules	34(82.9%)	46(95.8%)	0.075
Focal fibrosis	24(58.5%)	24(50%)	0.421
Bulla	9(22%)	7(14.6%)	0.367
CT radiomics characteristics, number (%)			
Interval distribution 1			0.008
[-950 ~ -850HU]	30(73.2%)	45(93.8%)	
[-850 ~ -750HU]	11(26.8%)	3(6.2%)	
Interval distribution 2			0.001
[-950 ~ -900HU]	7(17.1%)	24(50%)	
[-900 ~ -850HU]	34(82.9%)	24(50%)	

*CT-occult CLSCC and SEPL* CT-occult central lung squamous cell carcinoma and squamous epithelial precancerous lesions, *LC* lung cancer

### Independent Factors for the CT-occult CLSCC and SEPL

**Fig. 3:**
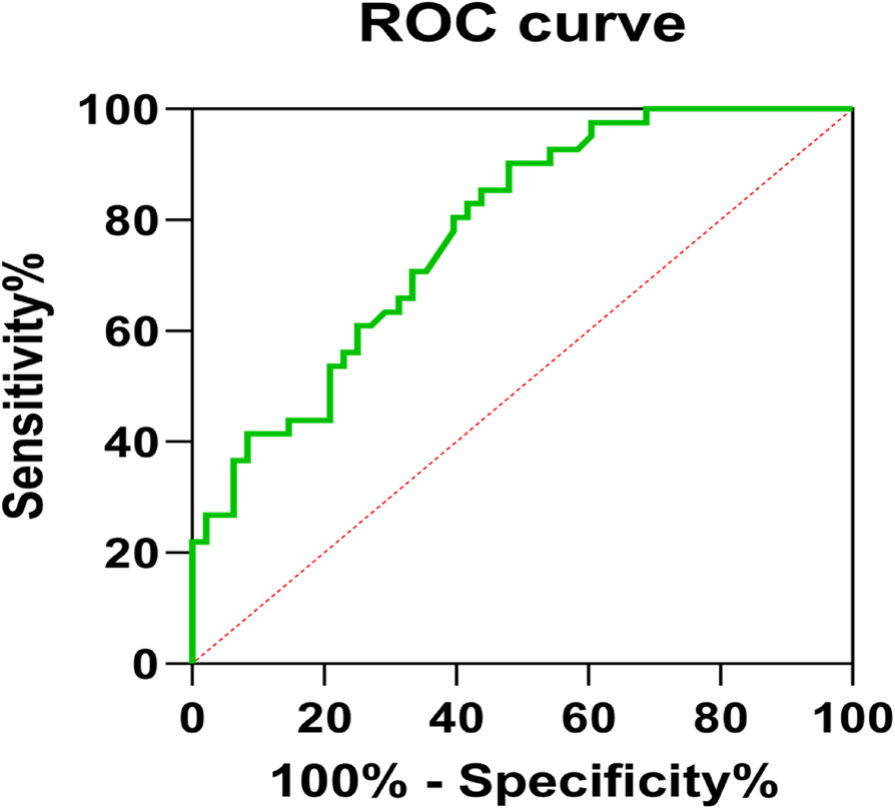
The ROC curve of the logistic model including 4 variables of pack-years > 20, CEA, Interval distribution 1 and Interval distribution 2 is shown, with an AUC of 0.776 (95% CI: 0.682–0.870). The sensitivity of this model was 90.2% and the specificity was 52.1%

We performed univariate and multivariate logistic regression analysis to explore independent factors for the CT-occult CLSCC and SEPL in the patients, which demonstrated that pack-years ≥ 20, in interval [-850 ~ -750HU] and in interval[-900 ~ -850HU] were independently associated with the CT-occult CLSCC and SEPL (Table 2). Through univariate screening, this study introduced 4 variables including history of heavy smoking, CEA and CT interval distribution 1 and 2, and built a multiple logistic regression analysis model. Ultimately, the logistic model obtained was statistically significant (*P* < 0.05) and the AUC value of the ROC curve drawn by the combined prediction probability value obtained reached 0.776, achieving certain prediction effect (Fig. 3).

**Table 2:** Univariate and multivariate analyses of independent factors for the presence of the CT-occult CLSCC and SEPL

Variable	Univariate Analyses		Multivariate Analyses	
	OR (95% CI)	*P* Value	OR (95% CI)	*P* Value
Pack-years ≥ 20	3.5(1.231 ~ 9.949)	0.019*	3.848(1.086 ~ 13.633)	0.037
CEA(ng/ml)	1.303(1.014 ~ 1.675)	0.039*		
CA125(U/ml)	1.027(0.986 ~ 1.069)	0.205		
CA199(U/ml)	1.011(0.982 ~ 1.041)	0.454		
Other nodules	0.211(0.041 ~ 1.081)	0.062		
Focal fibrosis	1.412(0.609 ~ 3.271)	0.421		
Bulla	1.647(0.554 ~ 4.902)	0.37		
Interval distribution 1	5.5(1.415 ~ 21.378)	0.014*	5.302(1.122 ~ 25.057)	0.035
[-950 ~ -850HU]				
[-850 ~ -750HU]				
Interval distribution 2	4.857(1.803 ~ 13.083)	0.002**	3.478(1.167 ~ 10.365)	0.025
[-950 ~ -900HU]				
[-900 ~ -850HU]				

*CT-occult CLSCC and SEPL* CT-occult central lung squamous cell carcinoma and squamous epithelial precancerous lesions, *LC* lung cancer

*P < 0.05

**P < 0.01

## Discussion

To our best knowledge, this is the first study to predict the occurrence of the CT-occult CLSCC and SEPL by analyzing the distribution changes in the number of pixels in the whole lung under different CT value based on middle-aged and elder Chinese male population.

Previous studies have shown that the 5-year survival rate of patients with stage I and Stage IV lung cancer has a huge difference, which is 55.5% and 5.3% respectively [16]. Meanwhile, there are more and more literature reports on central airway CT-occult lung cancer [17, 18]. Recently, the literature reports on central and peripheral double primary lung cancer with completely different pathological properties are also increasing year by year [19–23]. Therefore, the early diagnosis of central squamous cell carcinoma and squamous epithelial precancerous lesions is becoming increasingly important, as well as the screening of peripheral pulmonary nodules. Currently, the CT-occult CLSCC and SEPL can only be effectively diagnosed by bronchoscopy. Without bronchoscopic intervention, these mucosal epithelial lesions are first diagnosed only when they are visible on CT or when the lesions progress to the point where the patient presents relevant symptoms. Such a result is bound to seriously affect the treatment effect and survival rate of patients. In recent years, a number of studies on the use of bronchoscopy for lung cancer screening have been carried out worldwide, and the results show that bronchoscopy alone has limitations and cannot achieve the expected screening effect [24, 25]. However, relevant studies have also shown that bronchoscopy has high clinical practical value in the early diagnosis of central airway bronchial mucosal epithelial lesions [26]. At present, the Chinese Medical Association Lung Cancer Clinical Diagnosis and Treatment Guidelines (2022 edition) also recommends further bronchoscopy screening for heavy smokers [27], but there is no unified standard on how to accurately screen this population.

In this study, the research variable of the number of pixels under different CT values reflects the image feature of lung CT background sharpness. In the [-850 ~ -750HU] of interval distribution 1, the proportion of CT-occult lesions is more than that of no CT-occult lesions (26.8% Vs 6.2%, *P* = 0.008 < 0.05). In the interval distribution 2, we conduct a secondary segmentation of the interval [-950 ~ -850HU] with more pixel concentration in the interval distribution 1 and find that the pixels of the cases with the CT-occult lesions is significantly more in the [-900 ~ -850HU] than [-950 ~ -900HU] (82.9% Vs 17.1% *P* = 0.001 < 0.05). This study shows that with the appearance of CT-occult lesions, the total number of lung pixels tend to increase towards the region with high CT value, which is manifested as a decrease in lung background clarity in the subjective experience of CT images.

In this study, all patients have peripheral lung adenocarcinoma and most have a history of heavy smoking. The change of lung background clarity in CT images is closely related to the deposition of smoke particles. Relevant studies have shown that the composition and structure of cigarettes determine the size of inhaled smoke particles, and the deposition sites of different sizes of smoke in the lungs are different [28] and the entire lung tissue may be a potential area for cancer and tumor formation. Middle-aged and older men who smoke heavily are at risk of developing both squamous cell carcinoma and adenocarcinoma. Meanwhile, compared with adenocarcinoma, the risk of lung squamous cell carcinoma increases more rapidly with the increase of smoking time [29–31]. Therefore, single CT routine diagnosis of lung lesions can not fully meet the goal of early comprehensive screening of lung cancer. Although previous studies have shown that there is a certain degree of correlation between chronic lung diseases and the occurrence of lung cancer [32], the three CT signs introduced in this study have no significant value in the prediction of CT-occult lesions.

Although the sensitivity of the prediction model is 90.2%, its specificity is still low at 52.1%. The first reason for the low specificity of this study lies in the selection of patients, with low difference in baseline data and general clinical features between the two groups. All the patients in the two groups are middle-aged and elder male aged 49–70 years old. No acute inflammation and no COPD are found on lung CT, and no significant differences are found in the 3 common types of lung CT lesions. Second, although there is a difference in the history of heavy smoking between the two groups, the difference is not very large. The low difference in baseline data between the two groups is the significance of this study. If a control group of non-smoking female under the age of 50 are selected, the results will be very different. Although the specificity of this study is low, it has a high sensitivity to true positive cases. For such subjects, bronchoscopy is still necessary because of the objective existence of second-hand smoke exposure [33], even if they have no smoking history and even if there is a high false positive rate in this study.

Future studies will continue to be based on lung CT screening and extend the study to all populations (different genders and ages) undergoing screening. And then, more complete radiomic information will be introduced to analyze lung CT images. Finally, a predictive model is established to make a prospective prediction before bronchoscopy. Thus, accurate selection of risk groups can be realized.

## Conclusion

Although this study has such defects as small sample size, the researcher tries to balance the differences between the two groups in general clinical features and the selection of independent variables meet the requirements of lung cancer screening. Finally, in the CT value range [-950~-750HU], when the total number of lung pixels tend to increase towards the region with high CT value, the probability of the occurrence of CT-occult CLSCC and SEPL lesions also increases. Meanwhile, these results have guiding significance for the further study of radiomic.

## Data Availability

The datasets used and/or analysed during the current study are available from the corresponding author on reasonable request.

## Abbreviations

LC: Lung cancer
LDCT/CT: Low-dose computed tomography
CT-occult CLSCC and SEPL: CT-occult central lung squamous cell carcinoma and squamous epithelial precancerous lesions
COPD: Chronic obstructive pulmonary disease
ROI: Region of interest
IQR: Interquartile range
OR: Odds ratios
CIs: Confidence intervals
ROC: Receiver operating characteristic
AUC: Area under the curve
